# “Deeds not words”: the forgotten class

**DOI:** 10.1038/s41371-024-00935-0

**Published:** 2024-07-20

**Authors:** Bernadette Jenner

**Affiliations:** Division of Experimental Medicine, University of Cambridge, Addenbrooke’s Hospital, Cambridge, CB20QQ UK

**Keywords:** Clinical trials, Preventive medicine

## Abstract

The Sir Stanley Peart Essay Competition is an annual event run by the British and Irish Hypertension Society to encourage Early Career Researchers to continue the ethos of Sir Stanley Peart. Sir Stanley Peart was a clinician and clinical researcher who made a major contribution to our understanding of blood pressure regulation. He was the first to demonstrate the release of noradrenaline in response to sympathetic nerve stimulation. He was also the first to purify, and determine the structure of, angiotensin and he later isolated the enzyme, renin, and carried out many important investigations of the factors controlling its release in the body. This year, the essay topic was “Do we need new classes of antihypertensive drugs?”. In her prize-winning essay, “Deeds not words”: the forgotten class, Dr Jenner proposes that there is a need to address the unmet needs of hypertensive women, to increase their involvement in clinical trials and develop antihypertensives that are fit for purpose. Dr Jenner proposes that women are therefore the perfect class for new antihypertensives.

## Introduction

Hypertension remains the leading risk factor for early death and poor health worldwide [1] despite a plethora of inexpensive and effective antihypertensive therapies. Although hypertension was still considered an “essential” aspect to life prior to 1940s, since its identification as the most important modifiable risk factor for stroke, ischaemic heart disease and renal disease, the development of the perfect antihypertensive has remained elusive.

However, Women remain the forgotten class in hypertensive care. Despite raised blood pressure being the most important risk factor for death in women across the world, it is underestimated and insufficiently treated. Hypertensive guidelines set undifferentiated treatment thresholds at male-level risk, ignoring the increased risk of cardiovascular disease at a lower blood pressure level in women compared to men [2]. Even when hypertension is identified, current antihypertensive options are incompatible with a women’s lifelong health: unsuitable for pregnancy, under-researched for breastfeeding and consequential in menopause. The aetiology of hypertensive disorders of pregnancy, including pre-eclampsia and gestational hypertension, remains rudimentary with current treatment remarkably unchanged since the discovery of magnesium sulphate by Horn in 1906. Before creating new classes of antihypertensives, we should create equality for women in hypertensive care. Research and development should focus on how current antihypertensive classes work in women, how to develop classes of antihypertensives that are safe for those who are pregnant or lactating and how to prevent or adequately treat hypertensive disorders of pregnancy, conditions that not only impact on the lifelong health of the woman but also imprints on the lifecoarse of her baby [3, 4].

## Out with the old, out with the new

The mainstay of hypertensive treatment is lifestyle modifications and pharmacotherapy, options of which have remained stagnant for the past 20 years. Initial antihypertensives including thiocyanates, ganglion blocking agents (mecamylamine, hexamethonium) and catecholamine depletors (Reserpine, Raulwolfia alkaloids) were hampered by a narrow therapeutic index along with intolerable side effects and limited effectiveness. Hypertension treatment was transformed in the 1950s with effective treatments including thiazide diuretics, spironolactone, hydralazine and methyldopa [5], of which the latter remain the few medications used for hypertension in pregnancy today.

The 1970s saw the introduction of alpha- and beta-blockers, calcium channel blockers (CCB) with angiotensin converting enzyme inhibitors (ACE-i) and angiotensin II receptor blockers (ARBs) following in the 1990s. Unfortunately, during this boom of antihypertensive drug development, the impact of the thalidomide tragedy meant that instead of enforcing research to ensure the safety of medications in women, women of childbearing potential were ostracised from clinical drug trials. The 1977 Food and Drug administration (FDA) policy excluded women of child-bearing potential from clinical trials in drug development, thus leaving clinicians to decide blindly the risks and benefits of off-label prescribing in this population. The use of post-surveillance monitoring as the cornerstone of investigating maternal and fetal outcomes of medications in pregnancy has inherent bias, often giving conflicting evidence that is littered with confounding variables. Sildenafil, originally developed for treatment of hypertension, caused the “Pfizer riser” side effect for which it was promptly remarketed. By excluding women in clinical drug trials, side effect profiles whether good or bad, and pharmacokinetic sex differences in women are often missed leading to increased post-licensing side effects and overtreatment of women [6]. Nonadherence is a significant obstructor to current cost-effective antihypertensive treatment and women are 3 times more likely to be nonadherent when compared to men [7]. This may be explained by differing side effects as women are more affected by hyponatremia, hypokalemia and arrhythmia during treatment with diuretics, oedema with dihydropyridine CCBs and cough with ACE-i [2]. The reversal of the FDA policy excluding women from clinical drug trials did not occur until 1993, but as evidenced by the detrimental exclusion of clinically vulnerable pregnant women from COVID trials in 2020, the effects of such policies very much linger [8]. It is poetic that thalidomide, used as an unstudied and unlicensed antiemetic in pregnancy, should cause such a long-lasting impact and dearth of drug research in women.

Novel antihypertensive classes include small interfering RNA (siRNA) against angiotensinogen, Neprilysin and aldosterone synthase inhibitors, non-steroidal mineralocorticoid receptor antagonists (MRA) and dual endothelin antagonists. Many new antihypertensive classes remain focussed on modulating blood pressure by blocking an aspect of the renin-angiotensin-aldosterone system (RAAS). Older antihypertensives that similarly targeted this system are incompatible with pregnancy or those planning pregnancy as they cause fetotoxicity including renal dysfunction, oligohydramnios, intrauterine growth restriction and death [9]. It seems a missed opportunity that newer antihypertensives are therefore equally restricted in pregnancy by the same mechanism of action. Endothelin-1 antagonists such as Bosentan, account for three of the ten “Pregnancy Prevention Programme (PPP)” enlisted drugs due to their teratogenic effect causing craniofacial malformations [10]. With one third of pregnancies in the UK being unplanned or ambivalent [11], it is paramount that new classes of antihypertensives target novel mechanisms that, from the outset, make them compatible with pregnancy.

Of the novel mechanistic targets, siRNA appear the most exciting for treatment in women as larger molecules are less likely to transfer across the placenta and off-target side effects are likely to be reduced. Furthermore real-world data from COVID-19 RNA vaccines were reassuringly efficacious in pregnant people without fetal or maternal harm [12]. siRNA targeting soluble FMS-like tyrosine kinase-1 (sFLT-1), an anti-angiogenic protein marker of pre-eclampsia, and hepatic angiotensinogen have already shown promise in pregnant and pre-eclampsia animal models [13, 14]. However, entrenched gender knowledge gaps in women’s health means robust models of the physiological changes in pregnancy are lacking and developing such medication to fruition will likely be prolonged.

## Antihypertensives for the female lifecourse

There is an inextricable link between hypertension and oestrogen and as such women who are post-menopausal or with oestrogen imbalances such those with polycystic ovarian syndrome, premature ovarian insufficiency or infertility are at increased risk of hypertension. Although hypertension prevalence increases with age in both men and women, it increases exponentially in women post-menopause with a subsequent greater prevalence of hypertension in women over the age of 60 [15]. Altered vascular function, increased inflammation, up-regulation of RAAS and the sympathetic nervous system because of oestrogen reduction, makes development of a one-size-fits-all class of antihypertensive within this group difficult, but with an ageing population the number affected will only increase.

The orphan of hypertensive treatment is pre-eclampsia and gestational hypertension which although first conceptualised by Hippocrates [16], the pathophysiology remains unknown.

Discovering a treatment therefore remains a moving target as knowledge glacially develops. Defective trophoblast invasion and incomplete spiral artery remodelling in the placenta make the placenta an instinctive therapeutic target, however this pathophysiology does not explain the maternal cardiovascular changes seen prior to placental development and long-term maternal cardiovascular impact of gestational hypertensive disorders [17]. Without a definitive treatment other than delivery and removal of the placenta, current treatment options include age-old antihypertensives such as labetalol, long-acting nifedipine, hydralazine and methyldopa [18]. New biomarkers of pre-eclampsia including placental growth factor (PlGF) and sFLt-1, are potentially modifiable with new classes of antihypertensives but these are likely downstream effects which do not underpin the pathological process. As the intrauterine environment impacts on the developing fetus [19], longer term maternal and fetal cardiovascular risk may be reduced if pre-eclampsia and gestational hypertension can be prevented, in addition to the added benefit of a term delivery compared to a pre-term premature delivery in the context of pre-eclampsia.

## Summary

“Deeds not words” are required to improve knowledge of current and novel antihypertensives in women to tackle the rising prevalence of this significant untreated cardiovascular risk factor. Although policies have been made to ensure women are an essential part of pharmaceutical drug discovery, such as the implementation of National Institute for Health’s Women’s health initiative, the impact on the recruitment of women in antihypertensive drug development remains limited. There are persistent concerns for women in drug development, especially the legalities, complexities and perceived increased risk when involving the pregnant population [20] but this is short sighted. Involvement is critical to ensure we develop antihypertensives that are fit for purpose in this population with specific attention to gestational hypertensive disorders given the long-term maternal and fetal impact. Women are therefore the perfect class for new antihypertensives.
